# Drug screening: zebrafish as a tool for studying epileptic-related chemical compounds

**DOI:** 10.1007/s13238-015-0206-9

**Published:** 2015-09-24

**Authors:** Sha-Sha Zhao, Yi-Liao Wang, Ming-Zhu Sun, Lu Lu, Ya-Nan Wang, Delaney Pfister, Jessica Lee, Xin Zhao, Xi-Zeng Feng, Lei Li

**Affiliations:** State Key Laboratory of Medicinal Chemical Biology, College of Life Sciences, Nankai University, Tianjin, 300071 China; Tianjin International Joint Academy of Biotechnology and Medicine, Tianjin, 300457 China; Institute of Robotics and Automatic Information Systems, Nankai University, Tianjin, 300071 China; Department of Biological Sciences, University of Notre Dame, Notre Dame, IN 46556 USA

**Dear Editor,**

Here we report findings of zebrafish behavior and development during drug screening. In developing zebrafish embryos, epileptic seizure-like locomotor defects can be induced by brief treatment with GABA_A_ receptor antagonist pentylenetetrazole (PTZ). Using a trajectory video tracking system, we recorded zebrafish movements and constituted convulsant-like locomotor behaviors after treatment with PTZ. This led to the development of the first 3-D trace map of zebrafish locomotor behaviors in response to drug treatment. In addition, we examined the dose-dependent rescues of the locomotor defects in PTZ-treated animals after treatment with anti-epileptic drugs, such as valproate (VPA). We also examined the effects of drug treatment on animal development, such as hatching and mortality. Together, this research sheds new light for the development of new strategies for high-throughput drug screens using zebrafish models.

Zebrafish maintain a great evolutionary proximity to mammalian species, and have been used as a model organism for studying vertebrate developmental biology, physiology, and human disease (Paw and Zon, 2000; Chen and Ekker, 2004). Complementing the existing laboratory animals, zebrafish also created a niche in the field of drug discovery (Kaufman et al., 2009; Laggner et al., 2011; Gut et al., 2013). Recent studies suggest that zebrafish is suitable for screening compounds that target the molecular and cellular pathways involved in human diseases, such as cancer, heart failure, metabolic dysfunction and neural degenerative disorders (Owens et al., 2008; Kitambi et al., 2009; Baraban et al., 2013). A large number of synthetic and natural compounds have been identified, which include those that facilitate the survival of dopaminergic cells, protect auditory hair cells, enhance angiogenesis in regenerating nerve tissues, or increase neural regeneration by promoting the activity of radial glial progenitor cells. Molecular and cellular analyses of the products from the previous screens revealed that some of the newly identified compounds hold the potential for therapeutic treatment of neurological disorders, such as epilepsy, ataxia, sensorineual deafness, or tubulopathy. Also, through the screens, a large number of compounds that target the cascade of neural signaling transduction have been identified, such as those that modulate the activity of Shh, IGF or TGFβ pathways (Chen et al., 2009; Yang et al., 2013).

Epilepsy is a central nervous system disorder resulting from excessive and hyper-synchronous electrical discharges of the nerve cells (Hortopan et al., 2010). In zebrafish, epileptic seizure-like behaviors can be induced by treatment with GABA_A_ receptor antagonists, such as PTZ (Baraban et al., 2007; Afrikanova et al., 2013). While PTZ efficiently induces epileptic seizure-like behaviors, the characteristics of such behaviors and the effects of PTZ treatment in animal development have not been thoroughly examined. We characterized the zebrafish locomotor behaviors in response to epilepsy-specific drug treatment using a trajectory video tracking system based on a frame differential method (Sonka et al., 1998). In the absence of external stimuli such as gentle touches, disturb of swimming water, or tap of the container, the zebrafish embryos display very little spontaneous movements. At 7 days post-fertilization (dpf), for example, during a 30-min recording period, at most times the embryos were still and positioned toward the wall of the container and stayed at the bottom of the container (Fig. 1A, Row 1; Fig. 1B). The embryos displayed only a few instances of spontaneous movement (Fig. 1C), which resulted in less than 10 cm of total swimming distance (Fig. 1C).

**Figure 1:**
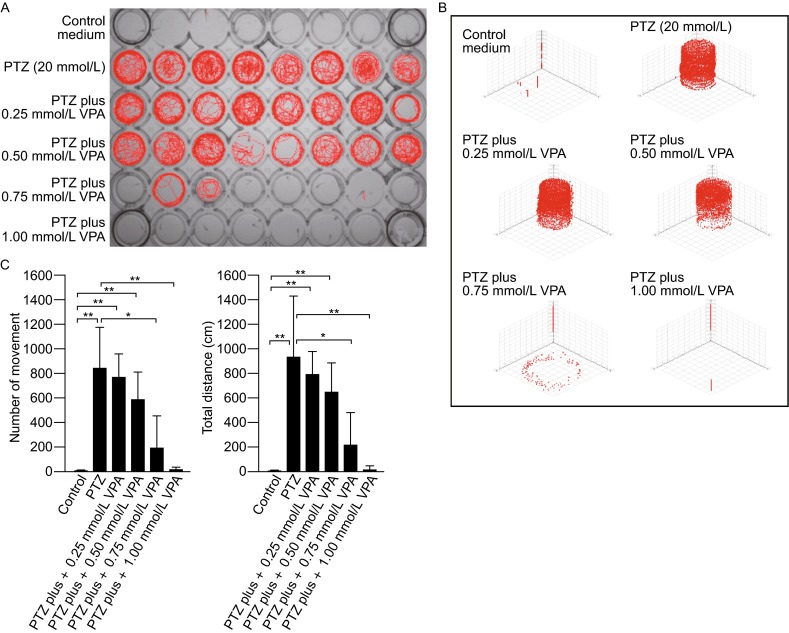
**Locomotor behaviors of control and drug-treated zebrafish embryos (7 dpf).** (A) Representative locomotor traces (red lines) of embryos during a 30-min recording period. The embryos were kept in individual wells in the 48-well plate, one embryo per well. Row 1, control embryos maintained in system-water; Row 2, PTZ- treated (20 mmol/L; treatment duration, 5 min) embryos; Row 3–6, PTZ (20 mmol/L) and VPA (in different concentrations) treated embryos. Note the increase in locomotor behaviors in response to PTZ treatment. The application of VPA resulted in dose-dependent rescues of the locomotor defects in PTZ-treated embryos. (B) Representative 3D traces (red lines) of locomotor behaviors recorded from a zebrafish embryo before and after drug treatment (recording period, 30 min). The embryo was kept in a well in the 48-well plate. In the control medium, the embryo spent most of the times at the bottom of the container and only displayed a few spontaneous movements. In response to PTZ treatment, the embryo became hyperactive, and swam in both the top and bottom half of the container. The application of VPA (0.25, 0.5, 0.75, and 1.0 mmol/L) produced dose-dependent rescues of the locomotor defects caused by PTZ. (C) Locomotor behaviors in control and drug treated embryos. Left: the number of spontaneous movement in control, PTZ, and PTZ + VPA treated embryos. Right: total swimming distances in control and drug-treated embryos. Data represents the mean ± SEM, *n* = 30; *P < 0.05; **P < 0.01

Treatment with PTZ (20 mmol/L; treatment time: 5 min) induced epileptic seizure-like locomotor behaviors, which include ictal, twitching, loss of posture, spiraling, and uncoordinated jerky movement. The embryos constantly moved, either along the wall of the container or crossed the middle of the container (Fig. 1A, Row 2). They were found in the bottom of the container as well as in the middle- and upper-part of the container (Fig. 1B). During a 30-min recording period, on average each embryo displayed 832.7 ± 339.7 instances of hyperactive movement, which resulted in 931.4 ± 509.4 cm of total swimming distance (Fig. 1C).

Treatment with VPA led to dose-dependent rescues of the hyperactive locomotor behaviors caused by PTZ. During the 30-min recording period, in response to a low concentration of VPA (e.g., 0.25 mmol/L), no significant changes in swimming patterns or total swimming distances were detected (Fig. 1A, Row 3; Fig. 1B; Fig.  1C). Along with the increase of VPA concentrations (0.50 mmol/L, 0.75 mmol/L), the locomotor defects caused by PTZ gradually diminished (Fig. 1A, Rows 4, 5; Fig. 1B; Fig.  1C). When the concentration of VPA increased to 1.0 mmol/L, the locomotor defects caused by PTZ were completely rescued. For example, at most times the embryos were still, similar as control embryos (Fig. 1A, Row 6; Fig. 1B), and the number of spontaneous locomotor movement and the total swimming distance were reduced to levels similar to control embryos (Fig. 1C).

To further investigate the effect of PTZ treatment on zebrafish development, we developed an assay based on membrane deformation in response to micropipette aspiration that is suitable for measuring the stiffness of the chorion membrane that surrounds the embryo before hatching (Fig. 2A). In control embryos, a linear pressure-length curve (P-L curve; aspirated pressure-membrane length) of chorion membrane can be recorded (Fig. 2B). Using this assay, we measured the shear modulus of the chorion membrane in response to PTZ treatment. The experiments were conducted in developing embryos treated with different concentrations of PTZ (10, 20, 30 and 40 mmol/L) or different treatment durations (between 1 and 7 h post-fertilization; hpf). Chorion membrane shear forces were measured at 24 hpf.

**Figure 2:**
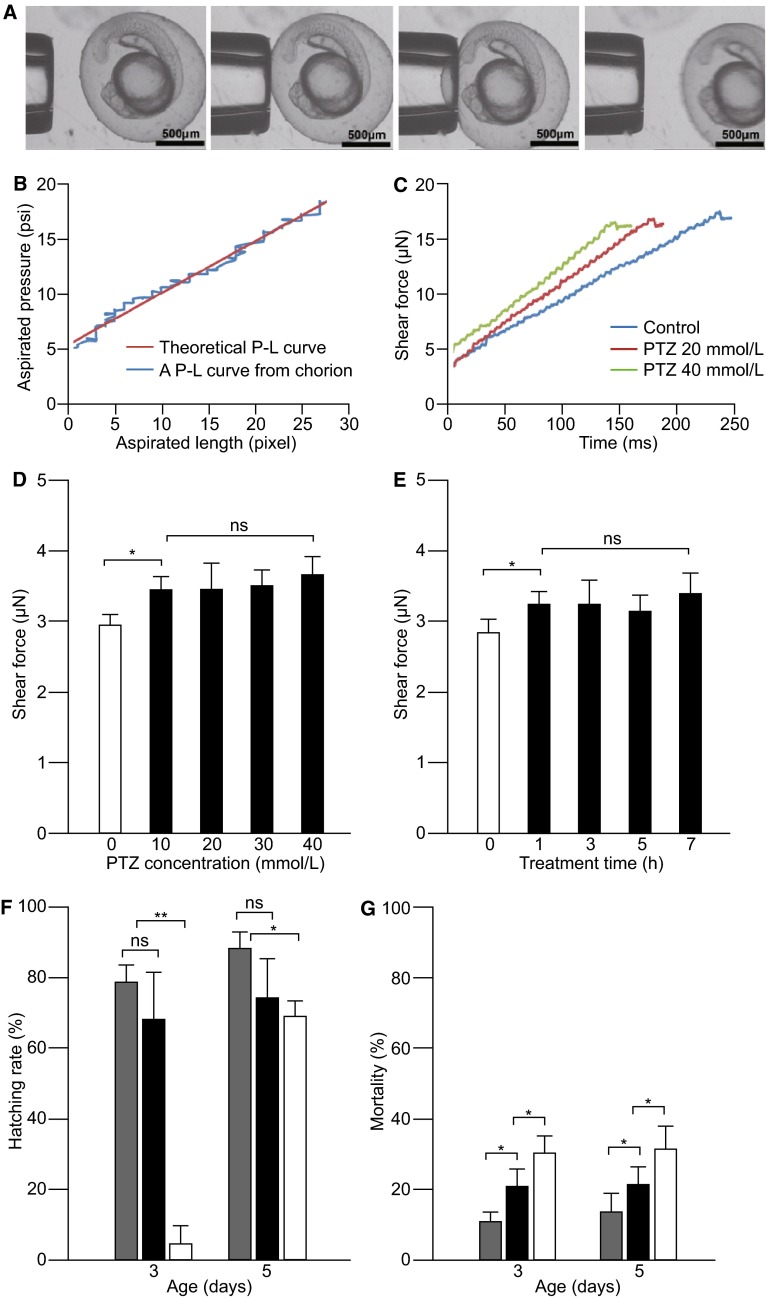
**Effects of PTZ treatment on zebrafish development.** (A) Diagrams that show the experimental set up for measuring the shear force of chorion membrane. From left to right: the pipette approached the embryo, attached to the chorion membrane, applied aspiration and recorded shear forces, withdrew from the chorion membrane. (B) Traces of theoretical and experimental P-L curves obtained from a 24-hpf control embryo. In both cases, linear P-L relationships were obtained. (C) Traces of chorion membrane shear forces in control and PTZ-treated embryos. Note the increase of membrane stiffness (decreases in time required to reach the maximum shear force) in response to PTZ treatment. (D) Chorion membrane shear forces recorded from zebrafish embryos (24 hpf) treated with different concentrations of PTZ (10, 20, 30 and 40 mmol/L). (E) Chorion membrane shear forces recorded from embryos (24 hpf) treated with 20 mmol/L PTZ for different durations (1, 3, 5 and 7 h). (F) Hatching rates at 3 and 5 dpf in control (grey bars) and PTZ-treated embryos (black and white bars). When PTZ was applied at 20 mmol/L (black bars), no significant decreases in the hatching rates were detected. When PTZ was applied at 40 mmol/L (white bars), the hatching rate was decreased in both 3 and 5 dpf embryos. (G) Mortality rates determined at 3 and 5 dpf in control (grey bars) and PTZ-treated embryos (black and white bars). In response to PTZ treatment (20 mmol/L, black bars; 40 mmol/L, white bars), significant increases in the death of the embryos were observed at both 3 and 5 dpf. Data represents the mean ± SEM, *n* = 30; *P < 0.05; **P < 0.01, ns: not significant

Treatment with PTZ increased the stiffness of the chorion membrane. In control embryos, the maximum shear force (membrane flexibility) was obtained approximately 240 ms after the onset of aspiration. The times that required to reach the maximum shear forces decreased to 177 ms and 149 ms, respectively, when PTZ was applied at 20 mmol/L and 40 mmol/L (Fig. 2C). In all cases (treated with different concentrations or different durations), the stiffness of the chorion membrane increased as compared to that recorded from control embryos (Fig. 2D and 2E). No obvious differences in membrane stiffness were observed when the embryos were treated using different PTZ concentrations (between 10 mmol/L and 40 mmol/L) or with different treatment durations (from 1 to 7 hpf) (Fig. 2D and 2E).

The increase of chorion stiffness due to PTZ treatment resulted in significant delays in hatching, particularly when PTZ was applied at high concentrations. In control animals kept in fresh system-water, at 3 days dpf, 78.6% ± 4.8% of the embryos hatched out of the chorion. By 5 dpf, 87.1% ± 4.9% of the embryos hatched (Fig. 2F). In response to PTZ treatment (20 mmol/L, 3 h of treatment between 3 and 6 hpf), no significant differences were observed in the hatching rate between the control and drug-treated embryos. When PTZ concentration was increased to 40 mmol/L, the hatching rate was decreased. For example, at 3 dpf only 5.0% ± 4.9% of the embryos hatched. When examined at 5 dpf, the hatching rate increased to 68.3% ± 4.8% (Fig. 2F), but still significantly lower than the hatching rate in control animals (*P* < 0.05). In response to PTZ treatment, the mortality of the embryos increased. In the control group, approximately 10%–13% of the embryos died when counted at 3 and 5 dpf (Fig. 2G). These included unfertilized eggs and embryos that had developmental defects. After PTZ treatment (20 mmol/L), the mortality rate increased to 20.8% ± 4.8% and 22% ± 5.0%, respectively, when examined at 3 and 5 dpf (Fig. 2G). Along with the increase of PTZ concentration (to 40 mmol/L), the mortality was further increased, i.e., to 30.1% ± 4.8% and 31.6% ± 6.7% when counted at 3 and 5 dpf (Fig. 2G). The embryos that survived PTZ treatment showed no obvious morphologic or locomotor defects in comparison to untreated control embryos.

In summary, zebrafish embryos display sophisticate locomotor behaviors in response to ambient stimuli and they are suitable for drug screens. Previously, the role of PTZ treatment on zebrafish neural activities (in the optic-tectum) and locomotor behaviors has been examined (Afrikanova et al., 2013). However, the characteristics of PTZ-induced locomotor defects, such as the frequency of convulsant-like movements, swimming patterns, dose-dependent rescues after VPA treatment, and the effects of PTZ treatment on animal development, remain to be determined. While this study did not investigate the mechanisms of epilepsy, it provided methods for studying the locomotor defects and development of zebrafish models with epilepsy. The locomotor trace maps generated by the trajectory video tracking system provide the first multi-dimensional (2D or 3D) views of the zebrafish locomotor behaviors in response to drug treatment. Instead of showing the representative locomotor characteristics collected at specific time points after drug treatment, the trace maps provide a tool for in vivo studies of drug effects on animal behaviors during the entire treatment period. It paves the way for functional analysis of the drugs on neural activity.

 The newly developed biophysical assay provides a tool for monitoring the toxicity levels of drug treatment on animal development. Specifically, it provides data that correlate the increase of chorion stiffness, delay of hatching, and the increase of mortality in response to drug treatment. Noted that when treated with different concentrations of PTZ or when treated with different time duration, no detectable differences in chorion membrane shear force were observed using our assays. Treatment with 20 mmol/L of PTZ did not significant affect the hatching, but increased the rate of mortality. With the increase of PTZ contraction (40 mmol/L), the hatching rate was decreased and the mortality was further increased. The delay of hatching may be attributed to the increase of animal mortality, i.e., due to decreased amount of oxygen or delay of food intake. It is possible that before hatching (approximately 3 dpf) the egg chorion acts like an effective barrier that protects the embryos from exposure to chemicals. From this point of view, it is conceivable to propose that for future drug screens, especially when the screens are conducted in zebrafish embryos during early developmental stages, de-chorination of the embryos may improve the result of the screens.

## Electronic supplementary material

Supplementary material 1 (PDF 251 kb)

## References

[CR1] Afrikanova T, Serruys AS, Buenafe OE, Clinckers R, Smolders I, de Witte PA, Crawford AD, Esguerra CV (2013). PLoS One.

[CR2] Baraban SC, Dinday MT, Castro PA, Chege S, Guyenet S, Taylor MR (2007). Epilepsia.

[CR3] Baraban SC, Dinday MT, Hortopan GA (2013). Nat Commun.

[CR4] Chen E, Ekker SC (2004). Curr Pharm Biotechnol.

[CR5] Chen YH, Wang YH, Yu TH, Wu HJ, Pai CW (2009). Transgenic Res.

[CR6] Gut P, Baeza-Raja B, Andersson O, Hasenkamp L, Hsiao J, Hesselson D, Akassoglou K, Verdin E, Hirschey MD, Stainier DY (2013). Nat Chem Biol.

[CR7] Hortopan GA, Dinday MT, Baraban SC (2010). Dis Model Mech.

[CR8] Kaufman CK, White RM, Zon L (2009). Nat Protoc.

[CR9] Kitambi SS, McCulloch KJ, Peterson RT, Malicki JJ (2009). Mech Dev.

[CR10] Laggner C, Kokel D, Setola V, Tolia A, Lin H, Irwin JJ, Keiser MJ, Cheung CY, Minor DL, Roth BL, Peterson RT, Shoichet BK (2011). Nat Chem Biol.

[CR11] Owens KN, Santos F, Roberts B, Linbo T, Coffin AB, Knisely AJ, Simon JA, Rubel EW, Raible DW (2008). PLoS Genet.

[CR12] Paw BH, Zon LI (2000). Curr Opin Hematol.

[CR13] Sonka M, Hlavac V, Boyle R (1998). Image processing, analysis, and machine vision.

[CR15] Yang XJ, Chen GL, Yu SC, Xu C, Xin YH, Li TT, Shi Y, Gu A, Duan JJ, Qian C, Cui YH, Zhang X, Bian XW (2013). Int Immunopharmacol.

