# Supplementary material for: Drug screening: zebrafish as a tool for studying epileptic-related chemical compounds
Source: Protein Cell. 2015 Sep 24;6(11):853–7. doi: 10.1007/s13238-015-0206-9 (PMC4624677; doi:10.1007/s13238-015-0206-9)
Supplement: Supplementary file 1 — Supplementary material 1 (PDF 251 kb) [file 13238_2015_206_MOESM1_ESM.pdf]

## Materials and Methods

*Animals*

Zebrafish (*Danio rerio*) were maintained in re-circulating system-water (KCl 0.05g/L, $NaHCO_3$ 0.025g/L, NaCl 3.5g/L and $CaCl_2$ 0.1g/L, pH 7.0-7.2) heated to 28$^{o}$C. Breeding colonies (AB strain) were fed twice daily with freshly hatched brine shrimps. Embryos were obtained from pair-wised spawning females and males. Embryos were obtained from wild-type breading colonies (AB strain) and staged according to previously published protocols (Kimmel et al., 1995). All experiments were performed in accordance with the NIH guidelines and regulations.

*Drug treatment*

Methods for PTZ treatment (e.g., drug concentrations and treatment times) were similar as previously described (Baraban et al., 2005, 2007; Afrikanova et al., 2013). PTZ was dissolved in distilled water and stored at -20$^{o}$C (stock concentration: 200 mM). Prior to PTZ treatment, the embryos were transferred into Petri dishes containing fresh system-water. For behavioral studies, PTZ stock solutions were added to the Petri dish at the final concentration of 20 mM, and for shear modulus measurements, PTZ was applied at the final concentration of 10, 20, 30 or 40 mM. To induce epileptic seizure-like behaviors, the embryos (7 dpf) were exposed to PTZ for 5 min, and then washed and transferred to 48-well plate containing fresh system-water (one embryo per well, each well containing 1275μl of system-water). For shear modulus measurements, unhatched embryos were treated with PTZ up to 7 hours. After drug treatment, the embryos were removed from the drug solution, washed and transferred to Petri dishes containing fresh system-water. The embryos were examined in different developmental stages as specified.

VPA was dissolved in 1% DMSO in distilled water and stored at -20$^{o}$C (stock concentration: 20 mM). Prior to VPA treatment, the embryos (within 5 minutes after the completion of PTZ treatment) were transferred into individual wells of the 48-well plate (one embryo per well, each well containing 1275μl of system-water), and then VPA was added at the final concentration of 0.25, 0.50, 0.75 or 1.00 mM. The duration for VPA treatment was 30 minutes.

*Behavior analysis*

The locomotor behaviors of individual embryos were recorded using a video tracking system based on the frame differential method (Microvision MV-VS078FC; 30 frames per second)

(Sonka et al., 1998). To suppress background noises, all the images were disposed through the Gaussian filter. Binary images were obtained by subtracting the previous frames from late-captured frames. The minimum bounding rectangle (MBR) of the largest connected component was calculated and center of the MBR was referred to as the location of the embryo. If the largest connected component of the MBR was greater than the threshold area, which was set to approximately 75% of the body-area of the embryo, then the embryo was considered moved; otherwise the embryo was considered immobile. Statistical differences in MBRs were determined by ANOVA.

*Shear modulus measurement*

The stillness of egg chorions surrounding the zebrafish embryos was evaluated by measuring the pressure-aspirated length curves (P-L curves) through an aspiration pipette (Spector et al., 1996). Equation 1 showed the relationships between aspirated length Lt, the magnitude of aspiration pressure P, and shear modulus μ.

$$\mathrm{L}_t = \frac{\sqrt{2}\,p_p R R_i (1-\mathrm{v}^2)^{0.75}}{2\pi\mu h(1+v)k^{0.75}} \frac{\alpha[\alpha f(\alpha_i)-f(\alpha_o)]}{\alpha^2-1}$$

Where

$f(x)=-0.33x^2+0.43x$ , $k=\frac{1}{12}(\frac{h}{R})^2$ , $\alpha=\frac{R_o}{R_i}$ , $\alpha_i=\frac{R_i}{R}$ , $\alpha_o=\frac{R_o}{R}$

The shear modulus was acquired under individual test conditions when the experimental parameters were provided, such as the inner and outer radius of the pipette (inner radius, between 220 and 250 μm; outer radius, 500 μm), and the dimension of egg chorion (radius, between 500 and 600 μm).
